# Oxidation of Nb(110): atomic structure of the NbO layer and its influence on further oxidation

**DOI:** 10.1038/s41598-020-60508-2

**Published:** 2020-03-02

**Authors:** Kuanysh Zhussupbekov, Killian Walshe, Sergey I. Bozhko, Andrey Ionov, Karsten Fleischer, Emma Norton, Ainur Zhussupbekova, Valery Semenov, Igor V. Shvets, Brian Walls

**Affiliations:** 1 School of Physics and Centre for Research on Adaptive Nanostructures and Nanodevices (CRANN), Trinity College Dublin, Dublin 2 Dublin, Ireland; 2 Institute of Solid State Physics, Russian Academy of Sciences, Chernogolovka, Moscow District, 142432 Russia; 3 School of Physical Sciences, Dublin City University, Dublin 9 Dublin, Ireland

**Keywords:** Structure of solids and liquids, Surfaces, interfaces and thin films

## Abstract

NbO terminated Nb(110) and its oxidation are examined by scanning tunneling microscopy and spectroscopy (STS). The oxide structures are strongly influenced by the structural and electronic properties of the underlying NbO substrate. The NbO is terminated by one-dimensional few-nanometer nanocrystals, which form an ordered pattern. High-resolution STS measurements reveal that the nanocrystals and the regions between the nanocrystals exhibit different electronic characters. Low-dosage oxidation, sufficient for sub-monolayer coverage of the NbO, with subsequent UHV annealing results in the formation of resolved sub-nanometer clusters, positioned in-between the nanocrystals. Higher dosage oxidation results in the formation of a closed Nb_2_O_5−y_ layer, which is confirmed by X-ray photoelectron spectroscopy measurements. The pentoxide is amorphous at the atomic-scale. However, large scale (tens of nanometers) structures are observed with their symmetry matching that of the underlying nanocrystals.

## Introduction

The surfaces of both oxidised transition metals and transition metal oxides form complex phases and reconstructions. This is due to the number of oxidation states transition metals can adopt and the ease at which the surface composition can be altered by annealing in reducing or oxidising environments^1–6^. Understanding the oxidation process and the properties of these surfaces is critical for heterogeneous catalysis and heterostructure oxide growth.

Niobium has the highest superconducting transition temperatures (T_C_ = 9.2 K) of all the elements^7^ and finds application in superconducting radio frequency particle accelerators^8^, superconducting quantum interference devices (SQUIDs)^9^, infrared photodetectors^10^ and Cooper-pair transistors^11^. Niobium and/or niobium oxide takes a prominent place in the tunnel barriers of single electron transistors^12^ and Josephson junctions^13^. The presence of oxides clearly influence the tunneling characteristics in such devices. Niobium oxides find further relevance as catalysts^14^. For example, small amounts of Nb_2_O_5−y_ and NbO_2_ can increase the catalytic activity of transition metals in redox reactions^14^. Nb_2_O_5−y_ sees application as a dielectric in capacitors with conductive NbO acting as the electrode. Understanding the interaction of these niobium oxides is vitally important as dielectric breakdown sees the formation of high resistance NbO_2_ at the Nb_2_O_5−y_/NbO interface, which prevents device failure^15^.

Niobium surfaces readily oxidise and niobium’s superconductivity transition temperature drops by approximately 1 K per percent oxygen impurity^16^. Dissolved oxygen resides predominately in interstitial octahedral sites^17^. Recently it has been shown that oxygen can only be removed entirely at temperatures above 2400 °C^18^. Sputtering can remove oxygen from the surface region but annealing as low as 200 °C results in diffusion of dissolved oxygen to the surface^18^.

The nature of the oxides which form an interface with niobium depend on the niobium termination. The oxidation of (110) terminated and polycrystalline niobium initially see the formation of NbO^19–22^ and subsequently NbO_2_ and Nb_2_O_5−y_ at greater exposures^19–21,23–25^. In the case of Nb(001), NbO_2_ and Nb_2_O_5−y_ are observed^26^, but not NbO. In all cases pentoxide is concluded to be the terminating oxide furthest from the niobium metal. Annealing in vacuum reduces the oxide surfaces, Nb_2_O_5−y_ can be reduced to NbO_2_ at ~150 °C^26^, and NbO_2_ further to NbO at ~300 °C^24–26^. Temperatures in excess of 2000 °C are required to remove NbO^18,27,28^. Oxidised and subsequent high temperature vacuum annealed Nb(110)^27–29^ and Nb(001)^30–32^ are the only oxidised niobium terminations investigated by scanning tunneling microscopy (STM). Both show similarities with the formation of a regular array of one-dimensional NbO nanocrystals^27–29,31,33^ several nanometers in length.

NbO formed on the surface of Nb is extremely stable^27,28,31^ and limits further oxidation^21,22^. It can play a large role in tunneling characteristics of niobium. In addition, NbO will influence the structure of higher valence niobium oxides which form an interface with NbO. For these reasons and considering the lack of detailed surface studies of the niobium oxides, we have performed an *in-situ* study of NbO terminated Nb(110) and its oxidation. We present high resolution spectroscopy measurements of NbO terminated Nb(110): two distinct regions of different electronic character are observed. Subsequently this termination is oxidised at low and high dosage, resulting in sub-monolayer and several monolayer coverages, respectively. We will show the structural and electronic properties of the NbO substrate plays a pivotal role in the initial stages of the oxidation process.

## Results

### NbO terminated Nb(110)

**Figure 1:**
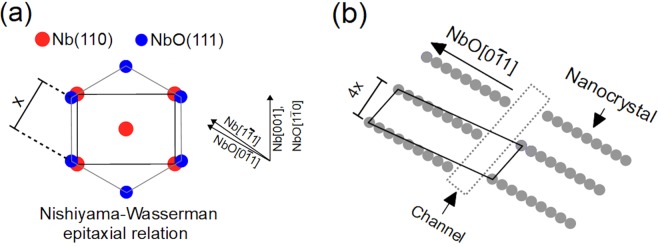
(**a**) The Nishiyama-Wassermann epitaxial relationship between the Nb(110) and NbO(111) lattices. The schematic depicts the unit cells and not all the atoms therein. The Nb[001] aligns with one of the NbO<110> directions ([110] is chosen in the schematic). The other two NbO<110> directions are rotated by 5^°^ with respect to the two Nb<111> directions of the Nb(110) plane. It is these two NbO directions which are the orientation of the nanocrystals, one of which is depicted in (**b**). (**b**) depicts the Nb atoms which terminate the NbO nanocrystals. The depicted rhombus defines the arrangement of these nanocrystals. Slight variation in the length of and the distance between nanocrystals are commonly observed. The area separating stacks of parallel nanocrystals, termed a channel, is highlighted.

When oxidised Nb(110) is annealed in reducing conditions a NbO layer forms at the surface^24,27–29^. The (111) plane of the NbO is parallel to the (110) plane of the underlying niobium crystal. NbO terminated Nb(110) has been reported after annealing the crystal oxidised in ambient conditions^29^ but also after annealing a niobium crystal with a clean surface, in which case oxygen dissolved in the bulk diffuses to the surface^27,28,33^. The NbO(111) forms Nishiyama-Wassermann or Kurdjumov-Sachs epitaxial relation with the Nb(110)^34^. The Nishiyama-Wassermann dominants the literature^24,27,29,33^ and it is depicted in Fig. 1(a). The annealing temperature and oxidation environment are critically important in determining which epitaxy relationship is present^34^. In this work the Nishiyama-Wassermann epitaxial relationship is observed.

The terminating NbO layer is not complete as compared to its bulk NbO counterpart: it is characterised by the periodic arrangement of strictly parallel rows of atoms 2–3 nm in length. Henceforth these one-dimensional rows will be referred to as *nanocrystals*^28^. The nanocrystals are orientated along the two NbO<110> directions which are rotated by 5° with respect to the two Nb<111> directions of the Nb(110) plane. The nanocrystals and their relative arrangement is depicted in Fig. 1(b), only one of the two possible domains is depicted. Misfit dislocations result in variation in both the displacement between the nanocrystals and length of the nanocrystal.

**Figure 2:**
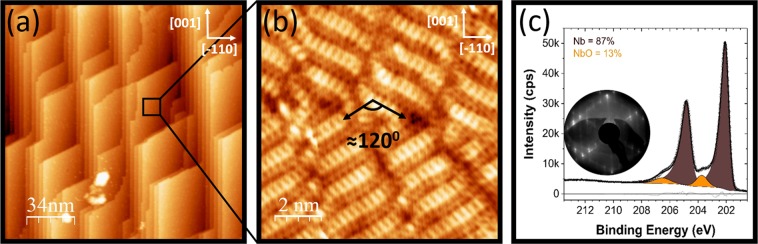
STM, LEED and XPS measurements of NbO terminated Nb(110). The ambient oxidised crystal was vacuum annealed at 850 °C prior to STM measurements. (**a**) Illustrates the terrace structure. (**b**) high resolution image illustrates the NbO(111)/Nb(110) nanocrystal termination. In (**b**) the two domains are evident. (**c**) Similar treatment during XPS measurements (700 °C in UHV for 20 mins) illustrate that the only oxide present is NbO. (**a**) 170 nm × 170 nm, *V* = 1.2 V and *I* = 74 pA. (**b**) 10 nm × 10 nm, *V* = 2 mV and *I* = 61 pA.

The niobium single crystal was introduced to the UHV chamber and annealed in a ultra-high vacuum (UHV) environment at 850 °C for 1 h. Figure 2 shows large scale and atomic resolution STM images of the surface subsequent to the annealing. The two domains of the nanocrystals of NbO terminated Nb(110) are evident in (b). The presence of the monoxide is further evidenced by XPS measurements depicted in Fig. 2(c), where NbO is the only oxide observed. Prior to the XPS measurements the crystal, which had been exposed to ambient conditions, was annealed at 700 °C in UHV for 20 mins. The long range order is demonstrated by low energy electron diffraction (LEED) measurements depicted in (c). The LEED is characterised by the superposition of a Nb(110) and a NbO(111) diffraction pattern, indicative of the Nishiyama-Wasserman epitaxial relation. The LEED produced by the Kurdjumov-Sachs epitaxial relationship would consist of the superposition of a Nb(110) and two NbO(111) diffraction patterns^34^. The diffraction pattern in (c) is qualitatively the same as the diffraction pattern obtained prior to STM measurements. These STM, XPS and LEED measurements support previous observations of this termination^24,27–29^.

**Figure 3:**
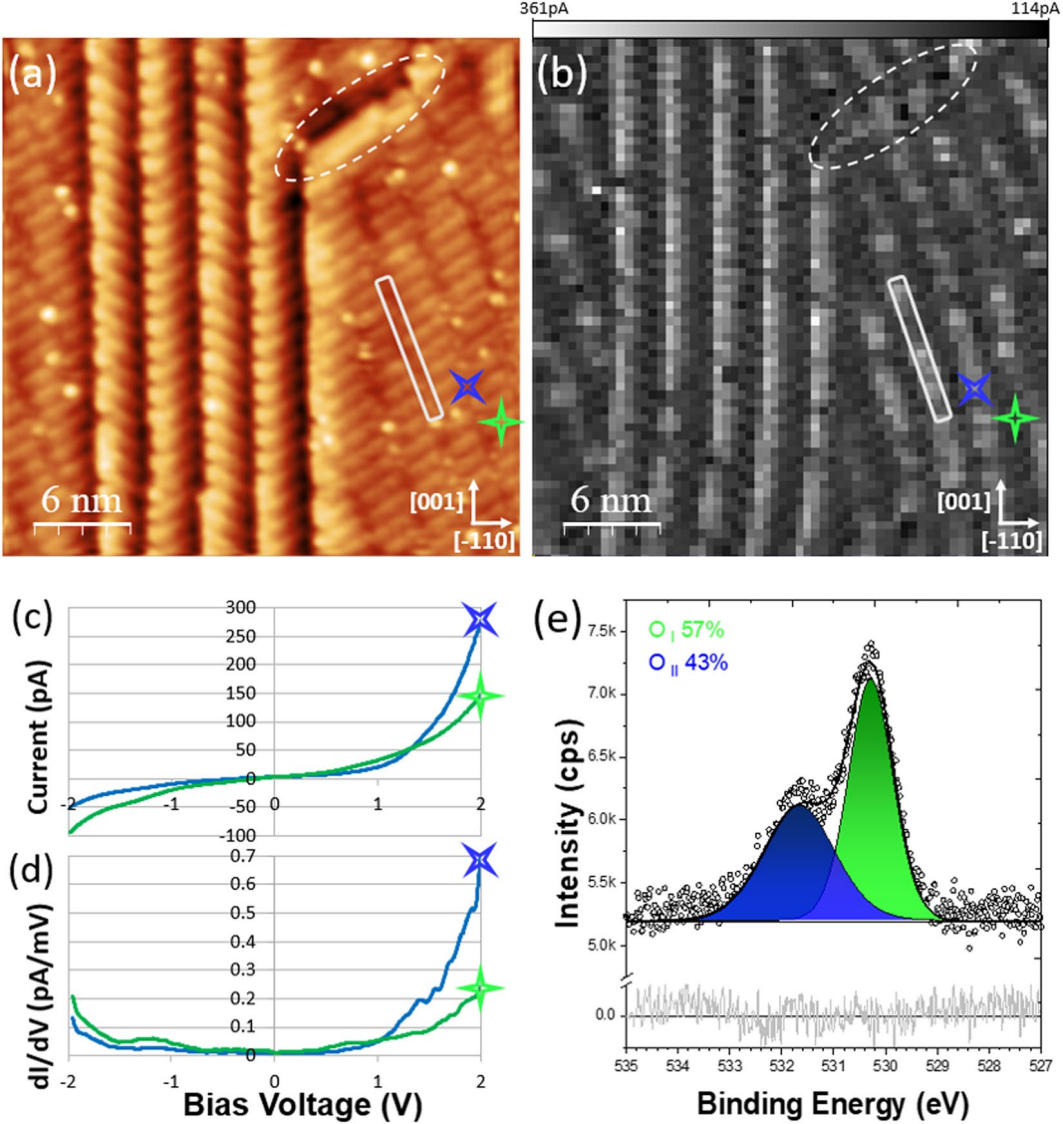
Grid spectroscopy of NbO terminated Nb(110). (**a**) (30 × 30 nm *V* = 0.3 V and *I* = 105 pA): STM image of the NbO(111)/Nb(110) nanocrystal termination. A grid spectroscopy measurement have been performed on the area in (**a**). (**b**) Depicts the tunneling current value (taken from the I(V)) at each spectroscopy point on the grid at a bias voltage value of 2 V. Bright lines (higher tunneling current) correspond to the terraces edges and the channels (an example of a channel is indicated by the white rectangle in (**a**) and (**b**)) in-between the NbO nanocrystals. (**c**,**d**) illustrate individual I(V) and dI(V)/dV spectra of two points on the grid highlighted by the green and blue stars in (**a**) and (**b**) positioned on a nanocrystal and a channel between the nanocrystals, respectively. The different electronic structure is most pronounced in the region between 1 and 2 eV above the Fermi level (+1 to +2 eV in the spectra). (**e**) O 1s spectra indicating the presence of two oxygen chemical states.

The STM image Fig. 3(a) contains a large terrace and a sequence of narrow terraces. The narrow terraces always have a width of ~3 nm in the [110] direction. In fact these narrow terraces are simply a stack of NbO nanocrystals. The orientation of the nanocrystals alternates step-by-step between the two domains. Grid spectroscopy measurements were performed on the area depicted in Fig. 3(a). 64 × 64 individual I(V) measurements were performed on the grid. At each point the spectra was averaged over 10 individual I(V)-curves. As the tip was moved across the grid, the tip height was stabilised by the constant-current mode scanning parameters. Prior to obtaining a spectroscopy measurement at any point, the feedback loop was turned off such that the current could be recorded as a function of the voltage while maintaining a constant tip height. Subsequently the feedback was turned on and the tip was moved to the next grid position. The constant-current mode scanning parameters used to move between points were *V* = 1.3 V and *I* = 50 pA. Therefore, for every I(V) measurement the current at 1.3 V was 50 pA. Variation in the tunneling current at different voltages are due to differences in the electronic structure. The voltage was swept between +2 V and −2 V. Figures 3(c,d) illustrate I(V) and dI(V)/dV (numerical derivative of I(V)) spectra of two points on the grid indicated by the green and the blue star positioned on a nanocrystal and in a channel between the nanocrystals, respectively. While the termination is conducting throughout, the two points are inequivalent. Most notable is the higher DOS between 1 and 2 eV above the Fermi level (+1 to +2 eV in the spectra). To illustrate this point Fig. 3(b) depicts the tunneling current from the I(V) at each spectroscopy point at a bias voltage value of 2 V. Brighter points corresponds to a higher tunneling current value at 2 V. This current map is characterised by bright lines corresponding to the terraces edges and in the channels in-between the nanocrystals on the terrace. The white rectangles in Fig. 3(a,b) highlight the channels, which are also depicted schematically in Fig. 1(b). It is interesting to note that the terrace edge parallel to the nanocrystals (highlighted by the dashed ovals in Fig. 3(a,b)) does not exhibit as high DOS compared to the terraces which coincide with the edge of the nanocrystals. This indicates that the presence of the nanocyrstal edges are in part responsible for the high DOS (in this energetic range) of the terraces running in the [001] direction. This is understood in terms of relaxation of the misfit-induced strain, which is easier at the edge of the nanocrystal; Arfaoui *et al*. commented that a larger nanocrystal length could be stabilized at the edge of a terrace, likely due to the reduced strain^35^.

Analysis of the O 1s peak, depicted in Fig. 3(e), can provide insight into the different electronic character of the nanocrystals and channels. Two components, one at 530.3 eV (O_I_) and one at 531.7 eV (O_II_), indicate the presence of two nonequivalent oxygen chemical states. These measurements support those performed by Razinkin *et al*.^27^. The ratio of these two states (57% O_I_, 43% O_II_) can be compared to the ratio of the bright and dark areas on the grid STS. One can speculate that the differing surface electronic structure is related to the distribution of the two oxygen states. This is corroborated by Razinkin *et al*. who observed an increase in the O_I_/O_II_ ratio when the polar angle was reduced. This indicates that the “highest” features, the nanocrystals, have a higher O_I_/O_II_ ratio compared to underlying layers. This is in agreement with the STS in which the nanocrystals and channels, which are at different heights, exhibit different electronic character. This is suggested to correlate to the different O_I_/O_II_ ratio.

### Low dosage oxidation NbO terminated Nb(110)

**Figure 4:**
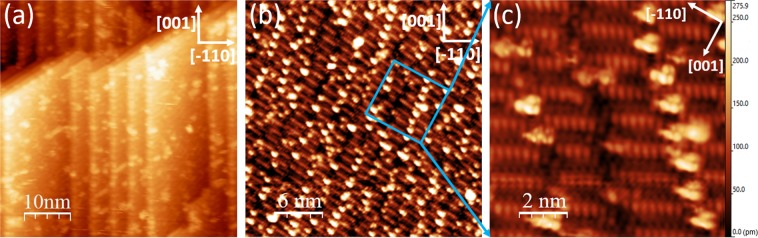
Low dosage oxidation of NbO terminated Nb(110). NbO terminated Nb(110) was exposed to a small dose (*P*${}_{{{\rm{O}}}_{2}}$ = 5 × 10^−10^ mbar for ≈30 s) of molecular oxygen at 77 K. The bright randomly positioned features in (**a**) are attributed to oxygen. Annealing the surface in (**a**) to 1400 °C results in the formation clusters ≈1 nm in diameter seen in (**b**) and (**c**), which form chains of up to 10 separated clusters. These clusters reside in between the niobium nanocrystals of NbO terminated Nb(110). High resolution STM image (**c**) demonstrates the features consist of 5 resolved atom-sized features. However, small variation in size and number of atom-sized features are observed. (**a**) 50 × 50 nm, *V* = 1.4 V and *I* = 68 pA. (**b**) 30 × 30 nm, *V* = 0.4 V and *I* = 66 pA. (**c**) 10 × 10 nm, *V* = 0.01 V and *I* = 62 pA.

In order to probe both the interaction of oxygen with this termination at the atomic-scale and this termination’s protective nature, NbO terminated Nb(110) was exposed to a low dose of molecular oxygen. Exposure was performed at liquid nitrogen temperature. The motivation to do so was to monitor the adsorption process in real-time with low-temperature STM. However, the STM tip was not stable in the oxygen environment even at very low oxygen partial pressure. NbO terminated Nb(110) was exposed to molecular oxygen (${P}_{{{\rm{O}}}_{2}}$ = 5 × 10^−10^ mbar, the base pressure was 2 × 10^−10^ mbar) at 77 K for 30 s. The STM image in Fig. 4(a) illustrates the presence of additional features on the surface as compared to the clean surface depicted in Fig. 3(a). These features, attributed to physisorbed oxygen, form some seemingly random shapes not correlated to the substrate. This surface with additional oxygen was annealed at 1250 °C for 30 mins and flashed at 1400 °C for 10 s in UHV. This results in the formation of clusters less than a nanometer in size, the vast majority of which reside in between the nanocrystals (Fig. 4(b)). Chains of up to 10 separated clusters are observed to form. A high resolution STM image of these clusters is presented in Fig. 4(c): the cluster is characterised by five distinguishable atoms-sized features. Small variations in terms of number of atoms and cluster shape are observed.

As was detailed in the previous section, NbO terminated Nb(110) forms upon UHV annealing either the oxidised (ambient or otherwise) surface or the oxygen-free surface which contains oxygen dissolved in the bulk. It is noted that annealing the crystal up to 1600 °C does not result in the formation of these clusters. Arfaoui *et al*.^28^ and Razinkin *et al*.^27^ also annealed the niobium crystal free of surface oxygen at 900–1900 °C and 1700 °C, respectively and observed the NbO(111)/Nb(110) nanocrystal termination identical to that in Fig. 2 without the presence of the clusters. It appears these clusters only form after the oxidation and subsequent annealing of the NbO(111)/Nb(110) nanocrystal termination.

These features are always observed in the channels in between the niobium nanocrystals. During heating, excess oxygen will likely desorb or diffuse to the channels where they are stable. Quite often we observe some poorly ordered areas in between the nanocrystals (for example see Fig. 2(b)), which was also observed by Arfaoui *et al*.^28^. Niobium residing in these disordered areas (or channels per the nomenclature of the text) and the mobile physisorped oxygen likely form niobium oxide compounds after high-temperature annealing. The formation of these features indicates the excess oxygen cannot diffuse through the NbO lattice to the underlying bulk niobium.

The grid spectroscopy of NbO terminated Nb(110) provides insight into the nucleation position of the clusters: 1–2 eV above the Fermi level the channels in between the nanocrystals exhibit a considerably larger DOS compared to the nanocrystals. At the current time it is unclear why the clusters reside in this specific electronic environment. However, a hypothesis is given by considering both the conductivity and enthalpy of the niobium oxides; The conductivity of the different niobium oxides reduces from metallic NbO to semiconducting NbO_2_ and finally insulating Nb_2_O_5_. In turn, the enthalpy of formation for NbO, NbO_2_ and Nb_2_O_5_ is − 415 kJ/mol, − 780 kJ/mol and −911 kJ/mol, respectively^36^. Therefore, the conductivity increases as the enthalpy decreases. One can hypothesise that the adsorbed oxygen will preferentially reside in the area of higher conductivity, which corresponds to a lower enthalpy of formation of an oxide, as is observed. The enthalpy also decreases as the oxidation state decreases. The model of Razinkin *et al*. concluded that oxygen in the channels has a lower oxidation state than oxygen in the nanocrystals^27^. It is noted that at negative bias voltage, the centre of the nanocrystals exhibit a slightly larger DOS than the channels.

Pulsing with the STM tip produces a very high electric field locally, which depends on the tip structure, tip-surface distance and the applied voltage. Pulsing can probe the stability of adsorbates and the energetic barrier for the transition of adsorbates from the surface to underlying layers^37,38^. We pulsed the clusters at ±7 V. This procedure leaves the clusters unaltered as judged by STM images before and after pulsing, further indicating their stability and the protective nature of NbO terminated Nb(110).

### High dosage oxidation of NbO terminated Nb(110)

**Figure 5:**
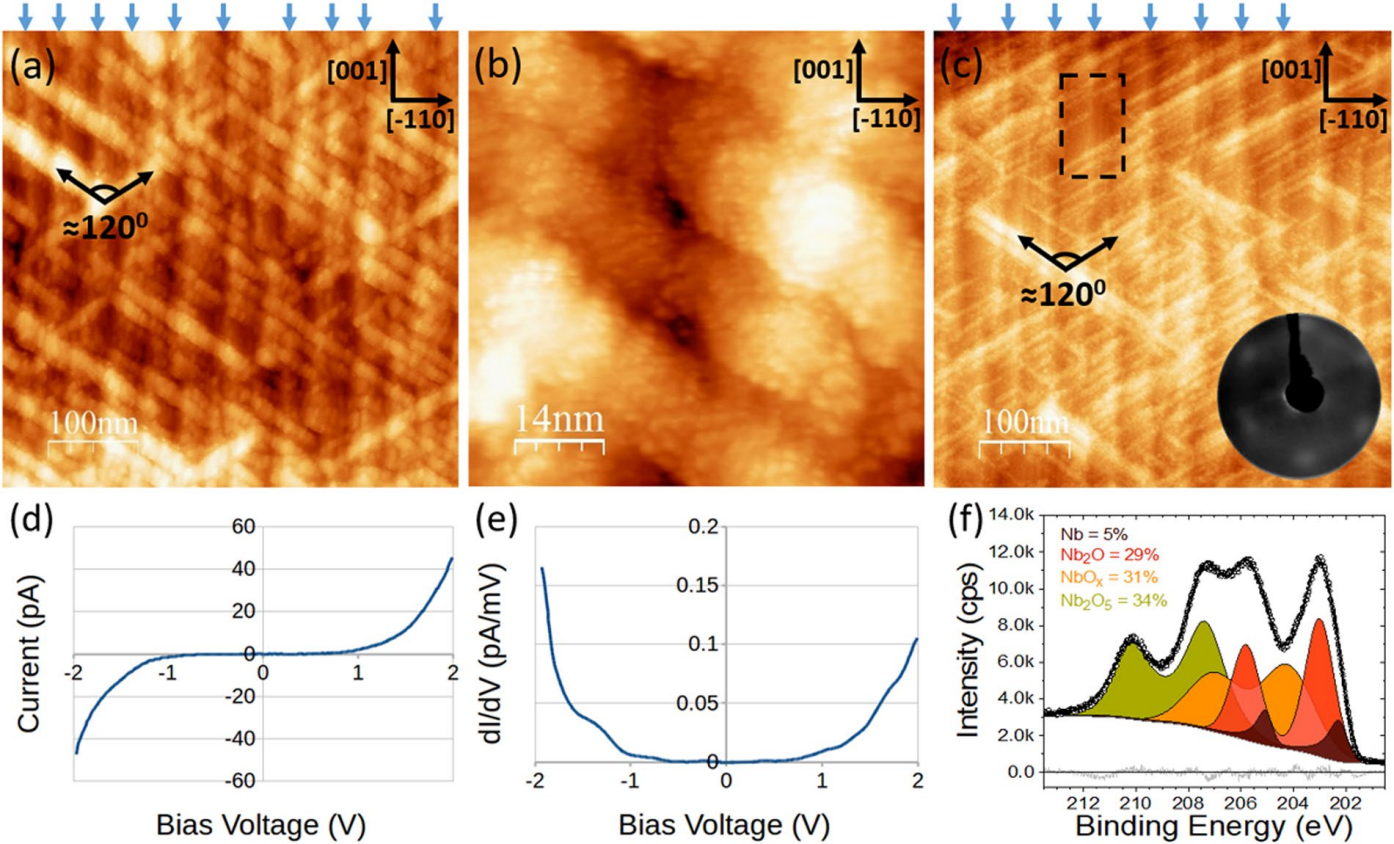
High dosage oxidation of NbO terminated Nb(110). (**a**) and (**b**): STM images after *in-situ* oxidation of Nb(110) single crystal at 600 °C for 10 mins at ${P}_{{{\rm{O}}}_{2}}$ = 2 × 10^−4^ mbar. Large scale (tens of nanometers) structure is observed in (**a**), while in (**b**) it is clear that there is no order on the nanometers scale. (**c**) STM image after gentle UHV annealing at 150 °C. The thickness of the oxide is reduced as evidenced by the appearance of terraces highlighted by the dashed square, which correspond to the underlying NbO. This is corroborated by the observation of LEED spots corresponding to the Nb(110) and/or the NbO(111). No diffraction spots were observed prior to this gentle annealing. The arrows at the top of (**a**) and (**c**) guide the eye to the terraces of the underlying NbO running in the [001] direction. (**d**) and (**e**) illustrates I(V) and dI(V)/dV spectroscopy measurements of the surface depicted in (**a**) and (**b**): the termination is semiconducting with a gap of ~1.5 eV. (**f**) XPS measurements have been performed on the crystal after a similar preparation procedure (700 °C anneal in a 2 × 10^−4^ mbar oxygen partial pressure for 1 h). The Nb 3d lineshape is comprised of metallic Nb, Nb_2_O, NbO_x_ and Nb_2_O_5_ contributions. (**a**) 500 × 500 nm, *V* = 1 V, *I* = 132 pA, (**b**) 70 × 70 nm, *V* = 1 V and *I* = 100 pA and (**c**) 500 × 500 nm, *V* = 1 V and *I* = 160 pA.

NbO terminated Nb(110) was annealed in an oxygen atmosphere (${P}_{{{\rm{O}}}_{2}}$ = 2 × 10^−4^ mbar) for 10 minutes at 600 °C. LEED measurements (not presented) indicated a disordered termination, with only a homogeneous background observed. STM measurements, depicted in Fig. 5(a), illustrate large scale structure characterised by features 50–500 nm in length and 10–30 nm in width. These features are observed in two directions separated by 120 ± 1°. XPS measurements have been performed on the crystal after a similar preparation procedure (700 °C UHV anneal and subsequent 700 °C anneal in a 2 × 10^−4^ mbar oxygen partial pressure for 1 h). The Nb 3d core level is depicted in Fig. 5(f). The lineshape has been fit with Nb(5%), Nb_2_O (29%), NbO_x_ (31%) and Nb_2_O_5_ (34%) components. The width of the NbO_x_ component suggests it is a disordered mix of NbO and NbO_2_, although it is closer to NbO than NbO_2_. LEED measurements recorded in conjunction with the XPS measurements were, as with the STM measurements, indicative of a disordered termination. The majority of XPS studies investigating the oxidation of niobium have focused on room temperature oxidation in air, which also results in the formation of several oxides including Nb_2_O_5−y_^22,24– 26,34^. All of those studies concluded that when pentoxide is present it terminates the crystal. The most notable study in this regard is the energy resolved 3d core-level spectra presented in M. Delheusy’s thesis, demonstrating that after oxidation pentoxide dominates the surface region^34^. Several studies have exposed the clean niobium surface to low partial pressures (10^−6^ − 10^−7^ mbar) of dry oxidation to study the oxidation process: niobium monoxide and dioxide first form and at larger exposures Nb_2_O_5−y_ forms on top^20,21^. With these points and the XPS in mind, the surface termination depicted in Fig. 5 is strongly suggested to be a thin-layer of Nb_2_O_5−y_.

Grid STS measurements were performed on a 15 nm × 15 nm area. The majority of points showed semiconducting character with a gap of 1.5 ± 0.1 eV. Although at some points the gap is observed to be as large as 2 eV. The growth of Nb_2_O_5−y_ is inhomogeneous^39^ and the resultant thickness variation can lead to a variation in the band gap at the surface. Bulk pentoxide has a band gap of 3.7 eV^40^ while other niobium oxides are conducting or have a small gap (NbO_2_)^41^. The difference between the measured band gap and bulk pentoxide’s bandgap can be due to the symmetry breaking surface, the interaction with the underlying lower valence oxides and/or varied stoichiometry due to the preparation procedure, which may induce a higher density of oxygen vacancies resulting in a higher density of Nb^4+^ and even Nb^3+^ sites^39^.

It is interesting that on the nanoscale (Fig. 5(b)) there is no ordering, however on the larger scale (tens of nanometers) we clearly observe ordering characterised by large features with distinct orientations. Considering the coherence length of 10–20 nm, LEED will only probe the structure within the features. LEED diffraction spots were not observed indicating the lack of atomic-scale order. Generally speaking, large scale order follows crystallisation and subsequent growth on a smaller length scale. However, here this is clearly not the case. We must consider the influence of the underlying niobium oxide structures and the parent Nb(110) substrate. The first point to note is that the orientation of these pentoxide features is the same as the nanocrystals of the NbO termination (see Fig. 2(b)). The directions correspond to the two NbO<110> directions of NbO(111) which are rotated by 5° with respect to the two Nb<111> directions of Nb(110) (see Fig. 1(a)). This NbO terminated the crystal prior to the oxidation and it is well established that in the case of heavily oxidised of Nb(110), NbO resides under the Nb_2_O_5−y_ layer^24–26^. In fact, the terraces of the NbO are visible even in the presence of the Nb_2_O_5−y_ termination, this is emphasised by the blue arrows above Fig. 5(a). Upon gentle *in-situ* UHV annealing at 150 °C, which is sufficiently high temperature to reduce the pentoxide thickness^26^, areas without any the pentoxide features are observed. In these regions the underlying terraces of the NbO structure are exposed. One such example is depicted by the dashed rectangle in Fig. 5(c). LEED measurements performed after the gentle anneal, presented inset of Fig. 5(c), show faint, broad diffraction spots corresponding to the Nb(110) substrate and/or the NbO(111) termination. With the reduced Nb_2_O_5−y_ thickness, LEED is more sensitive to these underlying structures. We propose that the NbO(111) acts as a seed for the initial growth and nucleation of the Nb_2_O_5−y_. It is noted that NbO terraces edges are in the same direction as the two nanocrystal directions, however terrace edges are also along the [001] direction but the pentoxide features are not observed in this direction. The NbO nanocrystal termination is dictated by the symmetry of the (110) termination of niobium, and hence, the Nb_2_O_5−y_ termination is, albeit indirectly, dictated by the specific termination of niobium. One can expect a modified pentoxide terminations when supported by other Nb terminations which would be an interesting avenue for further study. Pentoxide it is utilised for gas sensing^42,43^ and catalysis^14^. The unique structure observed here will very likely influence such processes.

## Conclusions

The NbO(111)/Nb(110) nanocrystal termination and its oxidation at low and high dosage has been examined. The contrasting oxides which form are strongly influenced by the substrates structural and electronic properties. High resolution grid scanning tunnelling spectroscopy measurements reveal the nanocrystals and regions between these nanocrystals exhibit different electronic properties, suggested to be linked to different oxidation states in the surface region identified by x-ray photoelectron spectroscopy. Low-dosage oxidation, resulting in sub-monolayer coverage, and subsequent ultra-high vacuum annealing of NbO terminated Nb(110) sees the formation of resolved clusters, which reside in between the nanocrystals in a region of distinct electronic character. The formation of these clusters, which are very likely niobium oxide in nature, illustrates the nano-porous nature of the NbO terminated Nb(110), which differs from the niobium bulk in which oxygen can diffuse easily. Annealing the NbO terminated Nb(110) in an oxidising environment (600 °C and *P*${}_{{{\rm{O}}}_{2}}$ = 2 × 10^−4^ mbar) results in a closed pentoxide termination confirmed by x-ray photoelectron spectroscopy measurements. No order is observed by low-energy electron diffraction or scanning tunneling microscopy on the scale of nanometers. However, structure on the scale of tens of nanometer is observed, with the orientation of the features coinciding with that of the nanocrystals. Scanning tunneling spectroscopy measurements demonstrate that this Nb_2_O_5−y_ termination is semiconducting with a gap of ~1.5 eV. This reduced gap relative to bulk pentoxide is suggested to be due to the influence of the underlying conducting niobium oxides. Furthermore, the relatively reducing conditions of the preparation may produce an under oxidised Nb_2_O_5−y_.

## Methods

Measurements were performed across two UHV systems, a STM system and a XPS system. The XPS and STM experiments were performed separately on the same (110) terminated niobium single crystal. The crystal was exposed to atmospheric conditions between experiments. Throughout this work the STM results were presented and the corresponding XPS measurements, obtained after applying similar preparation procedures, were qualitatively discussed. The microscope used in this work was a commercial low-temperature slider-type STM from Createc. All images presented were obtained in constant-current mode at 77 K. The STM tips used were [001]-oriented single-crystalline tungsten, which were electrochemically etched in NaOH. The bias is applied to the sample with respect to the tip. The sample temperature was measured from a K-type thermocouple up to 600 °C. Higher temperatures were estimated by an infrared optical pyrometer (*ε* = 0.25). XPS measurements have been performed using an Omicron MultiProbe XPS system using monochromated Al K_*α*_ X-rays (XM 1000, 1486.7 eV) with an instrumental resolution of 0.6 eV. Spectra have been analysed and fitted with CasaXPS. In order to remove contaminants the crystal was subjected to multiple sputter anneal cycles prior to the experiments.
